# Lupus Cerebritis as a Rare Neuropsychiatric Manifestation of Systemic Lupus Erythematosus

**DOI:** 10.7759/cureus.24973

**Published:** 2022-05-13

**Authors:** Ayola R Leitao, Molly S Jain, Enkhmaa Luvsannyam, Vanita Jayswal, Frederick Tiesenga

**Affiliations:** 1 Internal Medicine, Saint James School of Medicine, Park Ridge, USA; 2 Medicine, Saint James School of Medicine, Park Ridge, USA; 3 Surgery, Avalon University School of Medicine, Willemstad, CUW; 4 General Surgery, West Suburban Medical Center, Chicago, USA

**Keywords:** neuropsychiatric sle, autoimmune disease, lupus cerebritis, systemic lupus erythematosus, mixed connective tissue disorder

## Abstract

Mixed connective tissue disease (MCTD) is a rare autoimmune condition that shows an overlap of at least two connective tissue diseases (CTD) including systemic lupus erythematosus (SLE), rheumatoid arthritis (RA), scleroderma, Sjögren’s syndrome, polymyositis, or dermatomyositis. From a laboratory standpoint, MCTD is associated with high titers of anti-U1-RNP antibodies, which makes it difficult to determine whether it is a variant of each of the respective CTDs or a different entity altogether. Our objective is to report the case of a patient with MCTD presenting with status epilepticus who was ultimately diagnosed with lupus cerebritis. The case also highlights the development of complications unrelated to MCTD that made the management even more challenging. Overall, the authors emphasize the rareness of lupus cerebritis as a presentation, the diagnostic challenges faced due to the lack of classical manifestations of SLE, and how the complicated clinical course makes a downhill prognosis more likely.

## Introduction

Autoimmune connective tissue diseases (CTD) include five classical diseases: rheumatoid arthritis (RA), systemic lupus erythematosus (SLE), Sjogren’s syndrome, scleroderma, and myositis [1]. Mixed connective tissue disease (MCTD) is a rare systemic autoimmune disease and is referred to as “overlap syndrome” [1,2]. Patients with MCTD have at least two defined CTDs and a distinct antibody known as anti-U1-ribonucleoprotein (RNP) [2]. Alarcon-Segovia criterion (ASC) has been regularly used for diagnosing MCTD. Per ASC, to be diagnosed with MCTD, a patient should have a positive anti-U1-RNP antibody titer (>1:1600) as well as three out of the five following clinical features: Raynaud's phenomenon, hand swelling, sclerosis, synovitis, or myositis [2]. In 2019, a revised diagnostic criterion for MCTD was made in Japan and it included four requirements: Raynaud's phenomenon, presence of anti-U1-RNP antibody, organ involvement (including pulmonary arterial hypertension and aseptic meningitis), and an overlapping feature of either SLE, systemic sclerosis, polymyositis, or dermatomyositis [3].

SLE is a systemic disorder that can affect virtually any organ system in the body. Populations at increased risk of SLE include the African American community and women of reproductive age. Some of the common manifestations of SLE include cutaneous erythematosus/discoid lesions, photosensitivity, Libman-Sacks endocarditis, lymphadenopathy, lupus pneumonitis, and lupus nephritis [4,5]. Lupus cerebritis is a serious manifestation of neuropsychiatric SLE (NPSLE) that presents with neuropsychiatric symptoms [6]. The pathophysiology of NPSLE is yet to be fully elucidated [6]. Up to 80% of adults and 95% of children are affected by NPSLE; it can occur in the presence, as well as, in the absence of the active systemic disease [6]. Cognitive impairment, seizure, and psychosis are the most common symptoms of NPSLE and are associated with increased morbidity and mortality. Up to 40% of adult NPSLE symptoms present before or at the time of SLE diagnosis and 60% present within one year after the diagnosis [6]. 

We present a case of a patient with MCTD complicated by lupus cerebritis. Our patient required a tracheostomy during the hospital admission due to deteriorating pulmonary function. In addition, other clinical implications made treatment and management challenging.

## Case presentation

This is a case of a 48-year-old African American female who presented to the emergency department (ED) with status epilepticus and acute respiratory failure of unspecified origin. The patient’s past medical history was significant for mixed connective tissue overlap syndrome (with features of SLE, scleroderma, Sjogren's, and pyoderma gangrenosum), insulin-dependent type 2 diabetes mellitus, coronary artery disease, cerebrovascular accident without neurological deficits, deep vein thrombosis, hypertension, asthma, depression, and anxiety disorder. The patient’s past surgical history is notable for bilateral below-knee amputations and bilateral finger amputations secondary to complications of pyoderma gangrenosum and scleroderma respectively. Her social history is significant for remote use of nicotine and marijuana in her 30s. Her home medications include the following: albuterol 0.083% inhalational solution as needed, apixaban 5 mg, aspirin 81 mg, atorvastatin 80 mg, clonidine 0.1 mg, diazepam 5 mg, esomeprazole 40 mg, gabapentin 300 mg, hydroxychloroquine 200 mg, insulin lispro 100 units/mL, metoprolol tartrate 25 mg, oxycodone 5 mg as needed, prednisone 5 mg, and quetiapine 25 mg. 

**Figure 1:**
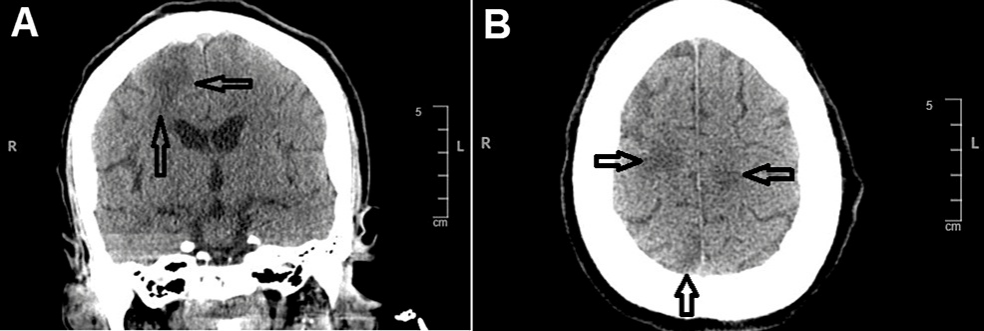
CT of the head without contrast displaying hypodensity in the right parietal cerebral cortex in coronal view (A) and the cerebral hemispheres in axial view (B).

In the ED, the patient was treated with phenytoin, levetiracetam, and intravenous methylprednisolone for seizure termination. She was intubated for respiratory support and airway protection. Physical examination revealed no active malar rash, discoid lesions, signs of joint inflammation, or serositis. Heart, lungs, and abdominal exams were also unremarkable. A renal workup revealed normal creatinine and glomerular filtration rate (GFR). Computerized tomography (CT) of the head without contrast displayed multiple areas of hypodensity in the cerebral lobes (Figure 1). 

**Figure 2:**
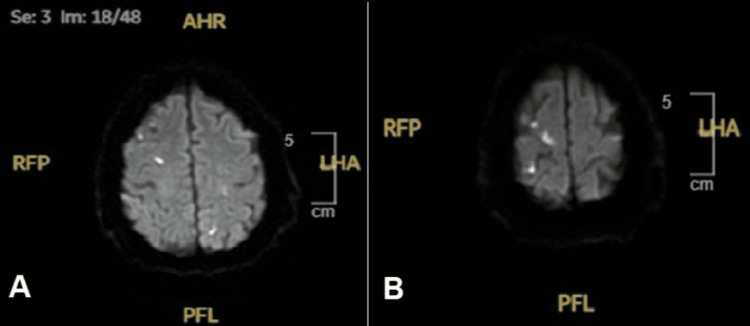
MRI of the brain without contrast exhibiting scattered foci of supratentorial ischemia (A) and left cerebellar ischemia (B).

Magnetic resonance imaging (MRI) of the brain without contrast exhibited scattered punctate foci of supratentorial and left cerebellar ischemia (Figure 2).

An electroencephalogram displayed epileptiform discharge consistent with the diffuse cerebellar process. Her plasma antinuclear antibodies (ANA) were positive. Lumbar puncture with cerebrospinal fluid (CSF) analysis revealed elevated anti-ribosomal P antibodies titers and undetectable levels of C3 and C4 complements. Additionally, laboratory findings revealed low C4 titers of 0.09 g/l and 0.15 g/l in the plasma and pleural fluid, respectively. All the above positive neurological and laboratory findings lead to the diagnosis of lupus cerebritis. Subsequently, the patient was initiated on 300 mg hydroxychloroquine and prednisone therapy (20 mg per day for one week, followed by 10 mg per day for one week, and 5 mg per day thereafter for maintenance). Other inpatient medications included anticoagulation with enoxaparin and an intense antiepileptic regimen with levetiracetam (500 mg twice daily) and phenytoin (50 mg twice daily).

The patient's respiratory failure was investigated with serial chest X-rays that displayed no evidence of infiltrates or lobar consolidations. CT scans of the chest and the cervical spine were non-contributary. Nasal swab cultures for methicillin-resistant Staphylococcus aureus (MRSA), influenza, respiratory syncytial virus (RSV), and COVID were negative as well. Persistent pulmonary complications included impaired spontaneous breathing and uncontrolled copious airway secretions, for which she received scheduled albuterol nebulization. Per neurology consultation, the only possible neurological etiology for the patient's decreased spontaneous breathing could be cervical spinal stenosis affecting the phrenic nerve. However, this differential could not be confirmed as MRI of the cervical spine was contraindicated in the patient due to an indwelling inferior vena cava (IVC) filter and a history of hip replacement. Finally, due to continued ventilator dependence and difficulty weaning the patient off the ventilator, a tracheostomy was performed to establish long-term ventilation two weeks after the date of inpatient admission. The patient also received a percutaneous endoscopic gastrostomy (PEG) tube for enteral nutrition.

While in the hospital, no further seizure episodes were reported. However, the patient's clinical course remained infested with multiple complications. She was diagnosed with urosepsis secondary to urinary tract infection and was treated with piperacillin/tazobactam and ceftriaxone sequentially. Following the resolution of urosepsis, the patient developed explosive diarrhea due to Clostridium difficile colitis and was managed with vancomycin. Other complications included decubitus ulcers, hypoalbuminemia, low-volume ascites (secondary to low albumin vs SLE), hypocortisolemia (likely secondary to chronic steroid use), anxiety, requirement of sedation, and physical restraints due to multiple attempts to dislodge life-supporting equipment by the patient.

In the current scenario, the patient is receiving supportive and symptomatic treatment in a skilled nursing facility. Treatment is focused on improving neurological sequelae with daily glucocorticoid therapy and antiepileptic therapy to ameliorate cerebritis and prevent seizure recurrence. Nevertheless, the prognosis remains very grim for the patient with a high rate of mortality. 

## Discussion

MCTD has a strong genetic association with HLA-DR4 and DR2 in relation to T-cell receptors and the generation of anti-U1-RNP [7]. In individuals with genetic predisposition, immune activation secondary to environmental factors is thought to play a role in the induction of MCTD [7]. These environmental factors include exposure to UV radiation, toxins, drugs, and chemicals [2,7]. The pathophysiological apoptotic mechanisms and molecular mimicry play a role in creating autoantigens and contribute to the process of MCTD [2]. The onset of MCTD can occur at any age with the average age of onset being 37 years [2]. Furthermore, MCTD does show a gender discretion with 75% of the patient population comprising women [2]. The symptoms of MCTD are non-specific and could include low-grade fever, arthralgias, myalgia, and malaise [2,7]. The prognosis of MCTD could also range from being mild and curable to life-threatening complications that could be fatal. Some of such complications include malignant hypertension, renal crisis, thrombotic thrombocytopenic purpura, cardiomyopathy, and arrhythmias [2,7]. Our patient presented with MCTD with a spectrum of disorders comprising SLE, scleroderma, Sjogren's syndrome, and pyoderma gangrenosum. Although she had mild symptoms for each of the aforementioned disorders, her major underlying complications were related to SLE.

Lupus cerebritis is a rare neuropsychiatric manifestation of SLE which can present with seizures, altered mental status, headache, anxiety, depression, psychosis, and pseudodementia [8]. It is a diagnosis of exclusion with complex etiology that could be attributable to various factors such as infections, drug use, brain abnormalities, and metabolic dysfunction [8]. Our patient also has an ongoing history of depression and anxiety. She subsequently presented with recurrent seizures further leading to an episode of status epilepticus that led to the severity of her overall health and hospitalization in ICU for months. The patient consequently developed acute respiratory distress due to severe muscle contractions secondary to status epilepticus which resulted in the need for endotracheal intubation for assisting with her airway needs. She was also operated on for the placement of a tracheostomy tube and a PEG tube for long-term ventilation and nutritional needs, respectively. Importantly, she was placed on various anti-epileptic drugs and glucocorticoids for the management and control of lupus cerebritis.

The highlight of this case pertains to MCTD and the way it can present with a focus on only one disorder symptomatically, which in our case was SLE. It is also evident in the presentation of lupus cerebritis, that it is a rare complication that requires further research. The individuality of this case also relates to how an autoimmune disease such as SLE can detrimentally and quickly impact the patient’s organs and overall health to the point of hospitalization, disability, and mortality. SLE and other autoimmune diseases require prompt workup and careful management with multiple specialists assisting with patient care. Follow-ups with a multidisciplinary team may aid in preventing complications or at least delay the progression of the disease.

Corticosteroids, cyclophosphamide, mycophenolate mofetil, azathioprine, rituximab, and abatacept are the most commonly used drugs in the management of lupus cerebritis. Immunosuppressants, hence, remain the cornerstone of the treatment [2]. The dosage and mode of administration (orally or intravenously), however, depends on the severity of the patient and the clinical judgment of the physician [2]. Treatment should be continued with taper being attempted only once the patient becomes clinically stable [2]. Importantly, in severe cases, where the patient is in a coma or if signs of cerebral edema or transverse myelitis are evidenced, plasmapheresis can be considered [2]. 

In general, lupus cerebritis is a very rare presentation of SLE or MCTD. Nevertheless, it needs to be included in the differential diagnosis for all patients with a history of MCTD presenting with seizures or the other neuropsychiatric manifestation of lupus cerebritis. Additionally, SLE workup including complete blood count (CBC), antinuclear antibodies (ANA) test, erythrocyte sedimentation rate (ESR), serum complement level, and renal biopsy need to be ordered immediately as this would promote earlier diagnosis and management of patients, thereby likely improving the overall prognosis.

## Conclusions

Autoimmune diseases can present with non-specific symptoms and their prognosis could range from mild to severe. MCTD is a significant autoimmune disease that may present initially with mild symptoms or could progress to develop serious manifestations. This was a rare case of lupus cerebritis, a neuropsychiatric manifestation of SLE, in a patient with previously diagnosed MCTD. It further highlights the importance of taking a detailed history in addition to accounting for the patient's medical and social history. Lupus cerebritis should be considered as a differential diagnosis in patients with previously diagnosed SLE who present with new-onset seizures, depression, or psychosis. In general, any patient with an autoimmune disease requires regular follow-ups, a prompt work up, and close monitoring to control the progress of underlying fatal complications. Finally, when individual patient needs are addressed, a multidisciplinary approach will provide a chance for a better outcome and quality of life.
